# Individuals in Collaborative Governance for Environmental Management

**DOI:** 10.1007/s00267-022-01693-w

**Published:** 2022-08-08

**Authors:** Julio César Zambrano-Gutiérrez, Laura Silvia Valente de Macedo, Marc Eric Barda Picavet, Jose Antonio Puppim de Oliveira

**Affiliations:** 1 School of Social Sciences and Technology, Department of Governance, Technical University of Munich, Munich, Germany; 2 Fundação Getulio Vargas São Paulo School of Business Administration (FGV EAESP), São Paulo, Brazil; 3 Institute of Advances Studies, Universidade de São Paulo (IEA/USP), São Paulo, Brazil; 4 Fundação Getulio Vargas (FGV EBAPE), São Paulo, Brazil; 5 Institute for Global Public Policy (IGPP), Fudan University, Shanghai, China

**Keywords:** Identity Theory, Waste Management, Urban Agriculture, Collaborative Governance, Pattern Matching, Florianópolis

## Abstract

Analyzing the effect of individual participants on collaborative governance processes in environmental management has been elusive due to lack of theoretical frameworks and data limitations. This study uses pattern matching to contrast identity theory with original data from 7 individuals participating in waste management and urban agriculture collaboration in Florianópolis, Brazil. What started as a self-organized initiative to manage an environmental problem, due to precarious waste management services, was scaled up to a citywide policy. Findings demonstrate that as the collaboration evolved over time, individual participants in municipal government transitioned between roles, organizations, and departments which affected their influence on the collaboration according to two transition styles: integrators (overlapping different roles) and segmenters (aligning roles with contexts without ambiguity). While the integrator-style participants were key to increasing sectoral diversity during the activation stage of the collaboration to produce innovative actions, segmenters contributed to formalizing the collaboration with appropriate institutional designs. However, the success of the collaboration after the institutionalization stage depended on the individual transition style and the power of municipal agents to have agency for influencing the collaboration. These findings have implications for adapting collaborative settings to respond to contextual changes that involve urban environmental issues.

## Introduction

Collaborative governance has been extensively explored in the literature to address environmental challenges since the 1990s (Gerlak et al. 2013). Typically, research focuses on the dynamics of collaboration regimes at the organizational level (Ansell and Gash 2007; Emerson et al. 2012; Bryson et al. 2015). However, scholars have long claimed that research about the role of individual actors participating in a collaborative setting needs further development (Bingham and O’Leary 2005). Our analytical framework follows the Ramarajan (2014) approach, in which identity can be self-defined, externally imposed based on roles, or a combination of both. Moreover, this research focuses on *role identity*, which is the cognitive stimulus for an individual to decide one’s appropriate behavior when enacting a role (Chreim et al. 2007; Jain et al. 2009).

As a result, an individual can have several identities based on his/her role in the organization and hierarchical position over time (see Table 1). In fact, there have been several attempts to understand how individuals’ role identities have an impact on public and private organizations over time but not on collaborative governance processes (Brower and Abolafia 1997; Ramarajan 2014; Kobarg et al. 2017; Luu et al. 2018; Tempelaar and Rosenkranz 2019; Mu et al. 2020). Moreover, while there have been recent efforts to disentangle collaborative governance processes throughout their life cycle (Douglas et al. 2020a; Douglas et al. 2020b; Torfing et al. 2020; Ulibarri et al. 2020), it is still unclear how individuals’ roles affect their identities and collaborative performance, particularly when individual participants in civil service have transitioned between roles, departments, and organizations during the life cycle of the collaboration. Based on the research gap identified above, the research question is: *How do participants’ role identity and transition style influence collaborative performance along the collaborative life cycle?*

**Table 1:** Key concepts based on identity theory

Concept	Definition	Example
Identity	Self-defined and/or externally imposed. Predisposition that motivates individual behavior.	Teacher, nurse, student, father, bureaucrat, activist.
Role	External behavior expectations. A specific set of activities with their own goals, values, beliefs, norms, and interaction styles.	Manager, Director, Coordinator.
Role identity	Identity is defined based on expectations and requirements for an individual holding a certain role.	“I am a nurse working as the Head of the Health Department, in which it is expected that I have good organizational and technical skills.”
Hierarchical position	Organizational positions are based on decision-making power.	Senior management, middle management, technical staff.
Role transition styles	Style of engagement in transitions between multiple roles.	(1) *Role integrators* engage relatively more on exploration instead of exploitation, overlapping different roles, (2) *Role segmenters* tilt towards exploitation instead of exploration.

Sources: Brower and Abolafia (1997); Chreim et al. (2007); Jain et al. (2009); Ramarajan (2014); Tempelaar and Rosenkranz (2019)

In order to respond to this question, this study looks empirically into a case where collaboration was key for starting and scaling-up an environmental management initiative, and actors had different roles during the collaborative life cycle. The case selected was a waste management and urban agriculture (UA) project in the municipality of Florianópolis, Santa Catarina State, Brazil, as the setting provides an analysis of key individual participants during the different developmental stages of the collaboration. The case analyzes a response to ineffective solid waste management, which in turn improved health conditions and provided food to vulnerable communities (de Macedo and de Oliveira 2021). Based on 19 targeted interviews and on archival data in official databases (e.g., municipal gazette publications; national database on academic careers) from 2008 to 2021, we identified how the role identities of key individual participants changed during the developmental stages of the collaboration and drove the implementation of waste management and UA initiatives. The collaboration resulted in benefits to the city, ranging from improving the quality of life of community members through organic waste segregation and collection for composting to establishing a regulatory framework that supports UA in schools and health centers throughout the city (Peixoto 2019; Abreu 2013; de Macedo and de Oliveira 2021).

Previous research identified how identity influences collaborative governance, but mostly by focusing on tensions between individual identity, derived from belonging to an organization, and collective identity, which originated from belonging to a collaboration (Thomson and Perry 2006; Bradley 2012; Kim 2016; Ran and Qi 2019; Lee and Esteve 2022). For example, Thomson and Perry (2006) pointed out how challenging it is for collaborative partners to manage their dual identities (i.e., individual and collective). This is because it is not always possible to reconcile self-interests from individual identities with the collective interests of the collaboration (collective identity). In fact, Ran and Qi (2019) proposed to study how power imbalances between partners reinforce focusing on the self-identity of the most powerful partner, to the detriment of collective identity, decreasing trust in the collaboration.

Tempelaar and Rosenkranz (2019)’s identity framework is particularly appealing to analyze transitions between multiple role identities as they propose to account for two types of role transition styles: (1) *integrators* or individuals who engage relatively more on exploration (i.e., acquiring new knowledge and developing skills) instead of exploitation (i.e., following organizational routines by using current knowledge), overlapping different roles because they do not impose boundaries between their multiple roles and (2) *segmenters* or individuals who tilt towards exploitation instead of exploration, differentiating each role, and aligning roles with contexts without ambiguity (Keller and Weibler 2015; Löwik et al. 2016; Tempelaar and Rosenkranz 2019). Hence, we argue that collaborative performance depends on how individual participants transition between their different role identities over time.

This study makes several contributions to better understand collaborative governance for environmental management (see Discussion section). First, it explores how particular aspects of individuals influence collaborative governance, as most of the research so far focuses on organizations. Second, it contributes to the theory of collaboration by scrutinizing individual role transitions over time to understand the enmeshed process of collaborative governance efforts and how transition styles influence collaboration in the different stages of collaborative development. Third, our research analyzes the role of power of individuals in the stages of the collaborative life cycle. Changes in decision-making power, embedded in new roles and hierarchical positions, determine whether individual participants have the agency to influence collaborative outputs and outcomes over time. Finally, we found that the combination of role identity and power is determinant for the fate of the collaborative regime after institutionalization, independent of the role transition style. Thus, the microfoundations explaining how individual identities based on organizational roles motivate individual behavior exhorts, at the very least, to consider potential role transitions of the actors participating in the collaboration as opportunities or threats to the sustainability of collaborations in response to contextual changes when addressing urban environmental issues.

## Collaborative Governance and Environmental Change

Strategies for dealing with environmental change have shifted from analyzing the barriers and opportunities for organizations to understanding collaborative efforts in dealing with complex environmental issues (Post and Altman 1994; Gerlak et al. 2013; Baird et al. 2016). There is mixed evidence and criticism regarding whether collaborative governance processes can overcome complex social issues derived from environmental issues, such as climate change, changes in hydrological systems and sources of fresh water, land degradation, and food systems (Gerlak et al. 2013; Scott 2015; Baird et al. 2016).

For instance, Scott (2015) shows evidence of improvement in water quality and habitat conditions on 357 watersheds in the United States when collaborative efforts are in charge of their management in comparison to watersheds that do not have an active collaborative group for their management. On the other hand, Baird Plummer and Bodin (2016)’s 1-year study on the evolution of a collaboration to co-manage climate change adaptation in the Niagara region of Canada showed that collaboration declined over time. Causes included low munificence of resources, lack of small wins or short-term goals achievements, and missing key actors in the collaboration. Thus, there is a need to further examine the components of collaborative processes, starting with the role of individual participants, to uncover the main factors influencing the productivity of collaborative governance over time (Emerson and Nabatchi 2015; Douglas et al. 2020a). To do so, this study focuses on individual participants in a collaborative effort to improve waste management and UA across stages of the life cycle of the collaboration. Due to space constraints, the following revision includes a summary of the theory and empirical findings associated with individuals in collaborative governance processes over time.

### Collaborative Governance Over Time

Collaborative governance is defined as “processes and structures of public policy decision making and management that engage people constructively across the boundaries of public agencies (…) in order to carry out a public purpose that could not otherwise be accomplished” (Emerson et al. 2012, p.2). Recent efforts to follow the trajectories of several collaborative governance initiatives have been possible through the analysis of the Collaborative Governance Case Databank (Douglas et al. 2020b). Key factors for successful collaborations include having strong incentives to join the collaboration, as well as achieving a high degree of institutionalization in the collaborative process (Douglas et al. 2020a).

Moreover, the agency of individual participants is critical, particularly in cases in which the structure of the collaboration is not ideal. In fact, Torfing et al. (2020) found that the role of institutional design, clear rules, and transparency of decision-making processes are not necessary to facilitate collaborative innovations involving diverse actors when strong leadership is present. However, institutionalization is key when collaborations lack leadership, mostly in the absence of leaders who can mediate conflict between diverse actors (Torfing et al. 2020).

The developmental stages during the life cycle of collaboration can be understood from both organizational and individual perspectives. On the one hand, theories from an organizational perspective have been the standard practice for studying collaborative governance (Lee and Hung 2021). In fact, seminal theoretical frameworks about collaborative governance are mainly based on the building blocks of organizational theory and organizational behavior to understand collaborative processes (e.g., principled engagement and shared motivation), structures of collaborations (e.g., institutional design), and their context (Ansell and Gash 2007; Emerson et al. 2012; Bryson et al. 2015). On the other hand, individuals are part of organizations, and their behavior determines the dynamics of a collaborative setting. As Champoux (2010) posits when referring to organizational theories: “The term organizational behavior is a little misleading, because it actually refers to the behavior of people in organizations—organizations themselves do not behave.” (Champoux 2010, p.6).

**Fig. 1:**
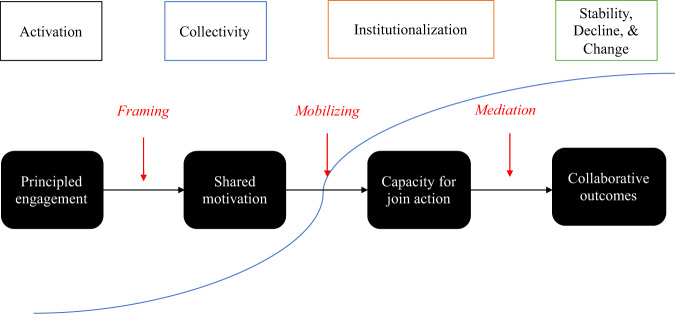
Life cycle model of collaborative partnerships from collaborative management and governance perspectives. Sources: McGuire (2002); Ansell and Gash (2007); Emerson and Nabatchi (2015); Imperial et al. (2016); Torfing et al. (2020); Ulibarri et al. (2020)

The interdependence between the structure of collaborative settings and the behavior of individuals is present in theoretical frameworks of inter-organizational collaboration. For example, Fig. 1 summarizes cumulative knowledge about key factors to improve the structure and process of collaboration. Individual participants need to have agency for framing the purpose of the collaboration to facilitate finding shared motivation despite different organizational goals. Also, individual participants need to have agency for mobilizing support not only from participating individuals but also from their organizations to join efforts and mediate conflict between diverse points of view to catalyzing innovative outcomes (see McGuire 2002; Torfing et al. 2020 for a reference).

### Understanding Individuals: Effects of Changing Roles, Departments, and Organizations In Collaborative Settings

In an attempt to explore individual participants’ attitudes and behaviors in collaborative governance, we propose to rely on research from other fields of the social sciences (e.g., psychology, sociology, general management) to understand the effect of individual participants on collaborative settings. There are at least three factors based on identity theory to help us understand individuals in collaborations: (1) the multiple roles that individual participants could have during a collaborative process, (2) the tensions between engaging in explorative and exploitative activities when transitioning between roles, and (3) the decision-making power embedded in each hierarchical position in the organization (Brower and Abolafia 1997; Ramarajan 2014; Tempelaar and Rosenkranz 2019).

First, individuals need to navigate multiple roles during their internal and external interactions at inter-departmental and inter-organizational levels, respectively. In fact, role conflict is a potential outcome due to the different demands, norms, beliefs, and values in a role and its inherent identity (Ashforth and Mael 1989). For example, Ashforth and Mael (1989) noticed how the Challenger disaster occurred when a senior engineer “helped to reverse a decision not to launch the Challenger when he was asked to (…) put on his management hat” (p.30).

Another application of identity theory is how an individual’s identity based on roles appears to tilt toward activities associated with exploitation or exploration (Tempelaar and Rosenkranz 2019). Individual ambidexterity has been understood in a continuum from *switching* between exploration and exploitation to *combining* both activities (Mu et al. 2020). Also, scholars understand exploration and exploitation from different perspectives. March (1991) links *exploration* activities in the organization with searching, taking risks, experimenting, playing, discovering, and innovating. Additionally, the author relates *exploitation* activities in the organization to implementing, executing, and producing. Other authors simplify the meaning of exploration as acquiring new knowledge and developing skills, while exploitation is about following organizational routines by using current knowledge (Keller and Weibler 2015; Löwik et al. 2016).

Role transitions can happen at the macro and micro levels (Ashforth Kreiner and Fugate 2000). Micro role transitions are recurrent transitions (e.g., work-home transitions), while macro role transitions are less frequent and could be permanent (e.g., retirement, promotion). While there are several theoretical perspectives to understanding multiple identities in organizational studies (see Ramarajan 2014 for a revision), this study uses a social psychological approach and focuses on macro role transitions to facilitate and understand the recollection of evidence regarding organizational role transitions (Ashforth et al. 2000; Ramarajan 2014; Tempelaar and Rosenkranz 2019).

Tempelaar and Rosenkranz (2019) identified two transition styles for managing multiple roles: (1) *segmenters* prefer to impose boundaries between their multiple roles to follow “behavioral blueprints” (p.1522) according to context; (2) *integrators* prefer to overlap roles searching for compatibility between the activities of multiple roles. Additionally, Tempelaar and Rosenkranz (2019) found evidence that individuals’ identities associated with the segmenter type have negative effects on individual ambidexterity: segmenters cannot combine exploitation and exploration activities while transitioning between their multiple roles.

While the Tempelaar and Rosenkranz approach can be useful to understand individual ambidexterity in private organizations, it overlooks the political factor. However, previous work in public administration has proposed that transitioning between multiple roles and identities is associated with the hierarchical position in the organization base on decision-making power (Brower and Abolafia 1997). In fact, Brower and Abolafia (1997) argue that bureaucratic positions with low hierarchy, based on their decision-making power, do not provide satisfactory identities to people. As a result, individuals with low-hierarchical roles engage in activities to search for roles that provide them status and respect through informal channels (deviations from organizational routines). Additionally, individuals with high-hierarchical roles base their identity on their formal position. Consequently, the achievement of organizational goals through formal channels (organizational routines) “becomes increasingly about self rather than about the organization” (Brower and Abolafia 1997, p.319). Table 1 summarizes the differences between identity, roles, role identity, hierarchical positions, and role transition style, which are key concepts in this study.

Finally, studies have recommended considering temporal and spatial dimensions when talking about ambidexterity (Bryson et al. 2015; Mu et al. 2020). For example, leadership roles in the activation stage are associated with actions to bring all relevant actors to the collaboration based on interdependence to achieve organizational and collaborative goals. However, during the phase of institutionalization, leaders concentrate on synthesizing and mediating actions to create the conditions for sustainable collaborative outcomes (McGuire 2002; Torfing 2016).

On the other hand, the spatial dimension in this study is related to individuals transitioning roles at different departments in the same organization (internal switching) and transitioning to different roles in another organization (external switching) *while participating in a collaborative setting*. This is important to understand the individual level of analysis since personality traits and individual preferences (e.g., economic motivations) are associated with organization switching (see Vinson et al. 2007; Frederiksen and Hansen 2017; Hansen 2014; McGinnis Johnson and Ng 2016; Piatak 2017). For example, Frederiksen and Hansen (2017) found that a wage increment of 5 percent is associated with switching managers from the public to the private sector. Interestingly, Vinson et al. (2007) present evidence that certain personality traits are related to organization switching. In fact, individuals with high scores in “extraversion” and “openness to experience” are associated with organization switching. While these personality traits are related to the *integrator* role transition style, individuals with a high score of “conscientiousness” are also associated with organization switching and the segmenter transition style.

Switching between departments inside an organization is also a source of individual identity based on power. In fact, power is unevenly distributed inside the subunits of an organization (see Salancik and Pfeffer 1974). As a result, individuals have agency in the collaboration according to the level of access and influence their department has. In fact, Imperial (1999) posits that power differences among participants increase coordination costs in the collaboration for two main reasons: (1) individual participants holding positions with a lack of power do not have the flexibility to implement agreements achieved in the collaboration, and (2) one or more participants with authority to take unilateral action can affect the sustainability of the collaboration due to lack of representation of the other individual participants’ interests. Consequently, it is expected that the effect of changes in role identities on the performance of the collaboration is conditional on the power embedded in individuals’ hierarchical positions. In fact, previous prepositions about identity and collaborative governance have pointed out how the collective identity of the collaboration is negatively affected in the presence of power imbalances between collaborative partners. In fact, the self-interest of the most powerful partner prevails, generating a lack of trust in the collaboration to achieve the collective purpose (Thomson and Perry 2006; Ran and Qi 2019).

## Methodology

We used a flexible pattern matching design in conjunction with a single case study for the empirical part of the paper. Pattern matching is an approach that reveals which assumptions and implicit mental models were implemented during research, helping readers to retrace the logical connections and conclusions made by the researchers (Sinkovics 2018). In practical terms, pattern matching is “the comparison of a predicted theoretical pattern with an observed empirical pattern” (Sinkovics 2018, p. 468). It typically begins with two paired investigations: a top-down, theory-driven investigation and a bottom-up, data-driven investigation. Patterns from the theoretical and observational domains are contrasted, and theory is extended or developed on this basis (Bouncken et al. 2021).

There are three categories of pattern matching: (1) full pattern matching, best suited to investigations whose goal is to examine causal relationships and based on a very rigorous research design; (2) flexible pattern matching, which includes techniques that provide greater flexibility for matching theoretical and observational pattern and most suited for exploratory studies; and (3) partial pattern matching, when only the top-down or the bottom-up phase is fully implemented (Sinkovics 2018).

Flexible pattern matching was adopted for this research, which combines deductive and inductive approaches in an exploratory investigation that builds on theory-driven research and allows for the emergence of data-driven insights (Sinkovics et al. 2019; Bouncken et al. 2021). The main objective of the top-down stage was to explore: *How do participants’ role identity and transition style influence collaborative performance along the collaborative life cycle?* From a comprehensive literature review, we deduced five expected patterns to answer the research question (Table 2). In the bottom-up stage, the expected patterns were confronted with empirical data from the case study to either validate^1^ or uncover unexpected dimensions (Sinkovics et al. 2019). As a result, we aim to contribute to the study of collaborative governance in new directions by including the individual participant perspective.

**Table 2:** Pattern Matching: the top-down approach

Dimension	Analytical framework	Expected patterns from theory	References
Type of role identity	Identity theory and bureaucratic politics	The transition between multiple roles is associated with the segmenter style when the new role includes a high position with decision-making power in the organization.	(Brower and Abolafia 1997; Ramarajan 2014; Tempelaar and Rosenkranz 2019).
		The transition between multiple roles is associated with the integrator style when the new role does not include a high position with decision-making power in the organization.	
Performance	Identity Theory and Collaborative Governance Theoretical Frameworks	Role identities associated with integrator style have positive effects to jump start collaborations.	(Emerson and Nabatchi 2015; Tempelaar and Rosenkranz 2019)
		Role identities associated with the segmenter style have positive effects on institutionalizing collaborations.	
	Identity Theory, Power, and Organizational Switching	New role identities as a result of organizational or departmental switching are associated with unstable post-institutional stages when individuals have limited agency for influencing collaborative performance.	(Salancik and Pfeffer 1974; Brower and Abolafia 1997; Imperial 1999; Thomson and Perry 2006;.Vinson et al. 2007; Imperial et al. 2016; Tempelaar and Rosenkranz 2019; Ran and Qi 2019)

The case of a waste management initiative in the municipality of Florianópolis in Brazil was chosen as various stakeholders changed their role identities, departments, and organizations along the collaborative trajectory, which moved from activation (small scale community project) to institutionalization (citywide initiative), and to a new phase with the pandemic (2020–2021), allowing the researchers to explore the different paths and impacts of the transitions.

### Data Collection

We compiled data through interviews and contrasted it with archival data (e.g., municipal gazette, national database for academic careers) as recommended by Guba and Lincoln (1982). Empirical evidence was obtained through semi-structured interviews conducted with people of interest working in or affected by the Florianópolis UA initiative. The research on the Florianópolis case was restricted to journal articles, dissertations, and gray literature, most of which in Portuguese (Abreu 2013; NSC Total 2016; Rede Semear 2016; Peixoto 2019; COMCAP 2021; Cultiva Floripa 2021; IBGE 2021; UNDP 2021; PMF 2022).

Due to the pandemic restrictions in 2020/2021, online conferences were held with city representatives to collect updated information and conduct semi-structured interviews with key stakeholders. Initial interviews were held with focal points selected by purposeful sampling and snowballing. Starting with the city’s focal point for ICLEI-Local Governments for Sustainability, senior and middle management civil servants were contacted, who in turn led to other key actors in civil society. A total of 19 individuals were interviewed between September 2020 and November 2021, including municipal staff and civil society representatives. Interviews followed a protocol designed by the research team members, lasting between 1:00 and 2:00 h; they were recorded and transcribed using Zoom, WhatsApp, Skype, Google, and Reshape software.

### Participants

Initially, we interviewed 19 individuals spanning 11 participating organizations to assess the context and background of the collaboration and to understand the individual roles during the collaboration life cycle. In Table 3, the interviewees are categorized according to their role transition pattern: (1) externally, in different organizations, or (2) internally, in different departments. Eight actors, including individuals that were not civil servants, kept the same role and organization. The remaining 11 underwent some transition, either in roles or organizations—internally by switching departments, or externally, moving to another municipal organization.

**Table 3:** Interviewees’ changes in roles and organizations

	Same role	Role transition
Internal switching (Same organization, another department)	14	**1**, **2**, **16**
External switching (Another Organization)	9, 10	15, **3**, **4**
Same Organization	7, 8, 11, 12, 13, 17, 18, 19	**5**, **6**

Numbers in each cell correspond to the Actor ID; the ones in bold are the focus of the study. For the sake of brevity, the selected actors in the table are numbered as follows: 1 Nurse; 2 Physician; 3 Politician; 4 Educator; 5 Appointee; 6 Manager; 16 Director

**Fig. 2:**
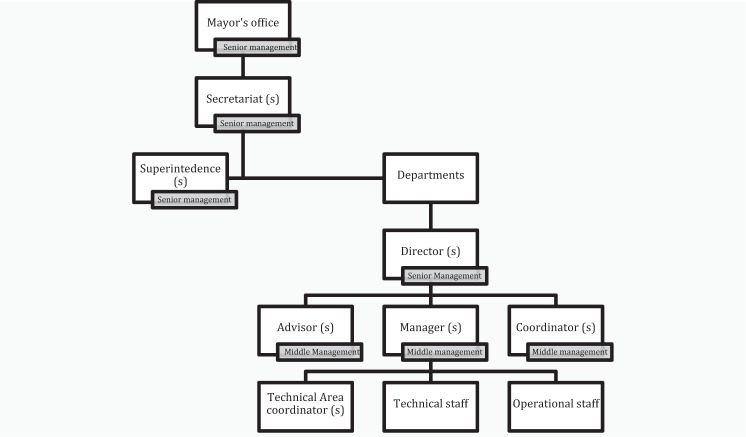
Florianopolis Municipality Chart modified

We prioritized the individuals that were directly involved in local government, as well as some of those in leadership roles engaged in the collaboration that have experienced a role transition. The categories related to hierarchical positions are based on the organizational chart of the city of Florianópolis (see Fig. 2). As a result, we selected seven respondents that underwent a role transition in government during the collaboration life cycle for our in-depth analysis. The sample was thus reduced to the following: four actors that transitioned between roles and switched organizations (externally) or departments (internally) in the municipality, and three that transitioned between roles in the same municipal organization between 2015 and 2021.

### Setting: Florianópolis Urban Agriculture and Waste Management

**Fig. 3:**
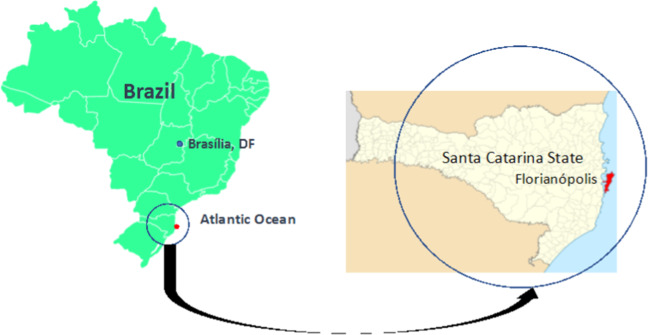
Florianópolis location in Santa Catarina state, Brazil. Sources: Ramos (2016) and Abreu (2006), adapted by authors

Florianópolis is a municipality and the capital of the State of Santa Catarina in southern Brazil. It is a touristic city located on an island (see Fig. 3) with approximately 508,000 inhabitants (IBGE 2021). Its Human Development Index (HDI) is 0.847, higher than Brazil’s (0.765) in 2019 (UNDP 2021). Nevertheless, Florianópolis displays high levels of inequality. In 2016, the municipality estimated that ~51,000 people lived in the city’s 64 *favelas* (slums) and that by 2028 this population would rise to over 76,000 people (NSC Total 2016).

Florianópolis is known for its excellence in community health care and quality nutrition in public schools. There are daycare units (DCUs) and community health centers (CHCs) in most neighborhoods, as well as good-quality public elementary schools. The municipality health system has pioneered integrating complementary and alternative medicine practices, such as acupuncture and phytotherapy, and offers internships for medical and nursing students. Nevertheless, providing these services to the poorer population is still lacking (de Macedo and de Oliveira 2021).

The Chico Mendes *favela* in the Monte Cristo neighborhood became well-known for the Bucket Revolution (*Revolução dos Baldinhos—RdB*), an innovative sociotechnical experiment to address precarious sanitation conditions. In 2008, as the community expanded, inadequate organic waste disposal and limited availability of municipal collection services led to a critical public health problem. Following the death of two residents due to leptospirosis transmitted by rats, a local movement supported by the community health staff assembled efforts to address the issue. With the help of a civil society organization (CSO) working with community agriculture (*Centro de Estudos e Promoção da Agricultura de Grupo*—CEPAGRO), residents managed to fundraise and organize a system to collect the organic waste separately, transforming it into fertilizer for local food production (Abreu 2013).

Besides the composting methodology itself, the collaboration between community members, other stakeholders, and the municipality was crucial to the initiative’s success. Despite significant limitations, the Bucket Revolution became a model of community waste management coupled with agroecological practices, garnering national and international support. Although much credit was attributed to social movements and the community (Abreu 2013, Rede Semear 2016), the municipality had a critical role in identifying the health issues, guiding, and supporting the neighborhood, and later on expanding the initiative to other areas of the city (de Macedo and de Oliveira 2021). Table 4 provides a timeline with key milestones, which will serve as the context to analyze the role of key individual participants in the section below.

**Table 4:** Timeline with key milestones for urban agriculture and waste management in florianópolis

	Activation							Institutionalization					Stability, Decline, Change
	2008	2009	2010	2011	2012	2013	2014	2015	2016	2017	2018	2019	2020
Context	Leptospirosis outbreak in Florianópolis							Dengue fever alert in Santa Catarina State	Municipal elections			Municipal elections	COVID Pandemic
Outputs	Mobilization led by CEPAGRO			RdB and CEPAGRO covenant				*Rede Semear* established	Urban agriculture intersectoral group established	Agroecology and Organics group established	Zero Waste Decree		Cultiva Floripa Decree
	RdB established			CEPAGRO and city covenant						Urban Agriculture Decree and PMAU	PMAPO law passed		Zero Waste 2030 Program

*RdB* Revolução dos Baldinhos (Bucket Revolution), *CEPAGRO* Centro de Estudos e Promoção da Agricultura de Grupo (NGO working with community agriculture), *PMAU* Urban Agriculture Policy, *PMAPO* Agroecology and Organics Production Policy, *Rede Semear* informal network including community and municipality members to implement urban agriculture and agroecology initiatives

## Findings

The in-depth analysis of seven individual participants provides the basis for the findings below. We identified their role and hierarchical position along the developmental stages of the collaboration. The data from the interviews helped us to determine exploitation and exploration activities associated with role identities and transition styles. Finally, we analyzed the impact on the organization, the collaboration, and the results of project scaling-up for each of the seven individuals in the three stages of the collaboration (see the details of each individual analysis in Annex 2). The observed patterns from the data and the theoretical implications are described below and summarized in Table 5.

**Table 5:** Flexible pattern matching summary

Expected patterns from theory	Examples of observed patterns from data	Implications for theory
The transition between multiple roles is associated with the segmenter style when the new role includes a high position with decision-making power in the organization.	The *physician* focused on *executing* the mission of his new department (i.e., data analysis) and became less engaged with his former colleagues and the UA network.	Incorporating the hierarchical position in the organization is a key factor in influencing the type of role transition style.
The transition between multiple roles is associated with the integrator style when the new role does not include a high position with decision-making power in the organization.	The *nurse* transferred *new knowledge* from volunteering in community gardening to innovative UA with herb gardens as prevention practices in the Family Health Program (PSF).	
Role identities associated with integrator style have positive effects to jump start collaborations.	The *politician* was a key activist in joining efforts between civil society organizations (e.g., CEPAGRO) and community members (e.g., Monte Cristo neighborhood) to promote UA policies in the city of Florianópolis.	Linking stages of development of collaborative governance and collaborative performance at the individual level of analysis.
Role identities associated with the segmenter style have positive effects on institutionalizing collaborations.	The *director* contributed to the development of the PMAU policy with her knowledge and by allowing her staff to be involved in UA to institutionalize the medicinal plant gardens in the community health centers (CHCs).	
New role identities as a result of organizational or departmental switching are positively related to unstable post-institutional stages when there is limited access to influence collaborative performance.	The *appointee* enacted organizational routines and amended policies to divide community-led UA initiatives from the municipal government focused on Zero Waste.	Linking the space (organizational switching) and time (stages of development) dimensions with the agency of individual participants on collaborative performance.

### Association Between Hierarchical Position, Role Identity, and Transition Style

#### Context

In 2015, civil society and the municipality joined to establish *Rede Semear*, a network on UA and agroecology that had a fundamental role in mobilizing concerned citizens (Rede Semear 2016). According to interviewees involved in the network, decision-making was shared between participants. For instance, the *Educator* stated that “In the past, we used to do everything together, it was a very horizontal management, all the members could give an opinion, they manifested themselves in all the referrals of the program management.” It was also acknowledged by all interviewees that ***municipal staff involved in developing the policy and implementing UA activities were enthusiastic and went above and beyond their professional duties to contribute to the collaboration****,* with knowledge sharing and off-the-clock time, for instance.

**Municipal officials and technical staff were key agents in taking the collaboration forward**, acting as catalyzers by playing multiple roles and often being personally involved, particularly through Rede Semear. This network was described by participants as a “horizontal” arrangement between civil society and the municipality. Decision-making about UA activities was shared between all members and implemented by the communities and the municipal managers engaged in the movement.

##### Nurse (Actor 1): Middle manager and integrator

She has worked in the health sector for 30 years, becoming a tenured civil servant as a nurse in Florianópolis’ Health Secretariat (SMS) in 2001, and being **promoted to program coordinator (middle manager) in 2015**. She has been a highly **motivated professional and has engaged in communal activities throughout her career** in Florianópolis. She became involved in the UA collaboration when a dengue fever alert in 2015 drove the city to launch a campaign to reduce the mosquito’s breeding sites, since there was a strong synergy with sanitation issues in the poorer communities. She was also **active in promoting medicinal herb gardens** in community health centers, daycare centers, and schools. She reported having often participated as a **volunteer in community gardening**. In the interview, she highlighted the importance of **sharing knowledge with staff from different organizations and departments**, and how people were committed to the project, going above and beyond their duties. She also emphasized the **importance of partners in the process**. “Whenever we detect an institutional weakness in one of our partners, we focus on supporting them.”

##### Physician (Actor 2): Senior manager and segmenter

He already had a senior management position and became director in Actor 1’s department. In 2017, due to changes in leadership, he was **moved to the Data Analysis Department**, **keeping his role as director** and continuing to participate in *Rede Semear* as an activist. During the pandemic, due to his opposing view on policies to address COVID-19 in general, **he took paid leave to finish his Ph.D., focusing on data management**.

##### Appointee (Actor 4): Senior management and segmenter

The political appointee is originally a career state-level civil servant (2004), who has been a **senior manager** in political appointments since 2017, **in different municipal departments** along the collaboration life cycle. He is an environmental engineer and administrator who specializes in solid waste management. Although he reported having previous involvement in participatory governance, **he shifted the administration towards concentrating decision-making about policies and budgets under his departments since he took office in 2020**. He contends that composting is an integral part of its municipal waste management policy, aligned with state and federal legal frameworks, including the National Solid Waste Management policy of 2010.

##### Educator (Actor 5): Middle management and integrator

The Educator is a biologist by training who joined civil service as an environmental educator in the Municipal Environment Foundation (FLORAM). She **became coordinator** and during the collaboration, she was actively involved in capacity building and training activities. FLORAM was **critical in establishing the collaboration between the communities and the municipality**. Her main interest was the collaboration’s multilevel and intersectoral governance model. **Through the collaboration, she continued her previous activities as an activist and educator in the city’s School Vegetable Gardens program**. She was a key player in the network through her role as a member of the city’s steering committee and in *Rede Semear* on behalf of the city.

### Association Between Role Identities, Hierarchical Position, and Collaborative Performance

#### Context

Florianópolis launched a **citywide program** on UA and sustainability, having waste management as the principal driver. The Urban Agriculture Municipal Program (**PMAU** in Portuguese) was established by decree in 2017 to foster organic food and medicinal plants production using natural compost, while diverting waste from the landfill. The PMAU is related to another decree on waste management, passed in 2018, establishing the Zero Waste 2030 program that includes composting and recycling measures. The PMAU involved organizing actions such as cleaning up, composting, and implementing community gardens.

By 2020, the city had mapped 117 community gardens in Municipal School Units, Health Centers, and neighborhoods. They are managed by volunteer community members and city staff, with technical support from civil society organizations (e.g., NGOs working with community agriculture), the state of Santa Catarina, and the Federal University of Santa Catarina (UFSC). Vacant public lots are also used as community gardens and composting facilities, thus avoiding waste dumping and contamination (COMCAP 2021).

Until early 2020, senior management had gradually increased the city’s role in systematizing the activities that were originally bottom-up, culminating in a new decree of July 2020 that **centralized administration and operations in the municipality**, without the participation of civil society representatives. It **amended the PMAU** to establish a steering group led by the city departments of agriculture, education, environment, and health, among others. The new decree also established *Forum Cultiva Floripa* as a consultative body, including civil society, to be convened by the steering committee. However, according to some of the original participants in the collaboration—volunteers and municipal staff, some of which act in their personal capacities -they were not consulted and learned about the changes in the municipal gazette. Furthermore, the **city government left** ***Rede Semear*, forbidding staff to participate on behalf of the municipality**.

After the new decree in 2020, municipal staff was not allowed to participate on behalf of the city, only in their personal capacities. As some interviewees reported, local politics became a barrier to UA implementation in communities. The Director, for example, acknowledges that “Political agreements end up somehow interfering. And speaking of this political issue, there are also some people who take it as a flag and use it in a political way, including in a partisan way, which ends up generating some internal barriers also for the group […] ends up creating a difficulty mainly for this to become a de facto public policy and not a political flag.” Likewise, the Appointee views **politics as a barrier to the PMAU implementation**, highlighting the controversy about changes in the original sociotechnical methodology and governance arrangements since the city became the leading agent: “There is a political movement [… *and*] **they think their participatory method is the only one that works**. So, this is the debate in Florianópolis. **They think there is no social participation because it was not their model**, so **we suffer this resistance**.”

##### Politician (Actor 3): Senior management and integrator

The Politician is a crucial player that galvanized community efforts to develop a sociotechnical experience acknowledged internationally. He joined the Agroecology movement as an activist at a very young age. After graduating in agriculture, he **became a trainer in an NGO to promote UA and agroecology as a strategy to address poverty and land tenure issues**. Together with a team of technicians and activists, he developed the participatory methodology to establish vegetable gardens for disadvantaged communities in derelict or abandoned public land. The collaborative governance experiment triggered many other initiatives and inspired cities nationwide. He was **elected as a city councilor** in 2016 and reelected in 2019 for another 4-year term in 2020. His trajectory shows that he was committed to the cause early on and **transitioned from being an activist to becoming a career politician**. He led the collective effort to establish the municipal network on UA and agroecology that **drafted the PMAPO bill** of law, passed in 2018. The agroecology network is perceived as a very politicized movement by municipal leadership, raising distrust and antagonism that affects UA initiatives overall. At the same time, being in a formal position as a legislator, he must act within an institutional framework that allows very little flexibility, in contrast with the informal networks whose interests he represents.

##### Appointee (Actor 4): Senior management and segmenter

The mini administrative reform in January 2020 strengthened the Municipal Environment Secretariat while splitting the solid waste management municipal autarchy (COMCAP in Portuguese standing for Companhia de Melhoramentos da Capital) into a policy-oriented superintendence and an operational department, facing strong opposition from workers and activists. With the pandemic, all selected waste collection was paralyzed. However, during the interview, the Appointee contended that **the reform enabled the structuring of new departments and the launching of the Zero Waste 2030 Program**, which was established in 2018. Besides concentrating the waste management budget under his department, **he fractured the participatory UA movement inside the municipality**, which was further harmed by the pandemic, **both in terms of power and budget**. Critics accuse the department of using funds that were allocated to the *Cultiva Floripa* program implementation to invest in equipment and contracts for the Zero Waste policy.

##### Educator (Actor 5): Middle management and integrator

Her **main concern for the municipal** ***Cultiva Floripa*** **policy regards funding**. She considers that there already are the necessary enabling environment and critical mass to support a strong program. However, due to the pandemic and silo-thinking in municipal administration, **her department was unable to include UA in its budget**. During the interview, she did not criticize the administration for prioritizing waste management and the Zero Waste program. However, she highlighted that the **changes in the governance structure of the PMAU had demobilized municipal staff involved in the collaboration since 2020**. The *Cultiva Floripa* program left the *Rede Semear* network, thus breaking the bond between city staff and community members.

##### Manager (Actor 6): Middle management and segmenter

She has been a career civil servant with the city since December 2012 and an **environmental manager** at COMCAP throughout the collaboration life cycle. Her role at the beginning of the collaboration was as a technical advisor to the director, and after the changes, she became a senior technical staff without involvement in the UA program. She acted as a supportive liaison between the collaboration participants and senior level management. Although she was not defined as an activist, she is perceived as supportive and competent. She considers *Cultiva Floripa* a successful collaborative governance initiative from her department’s perspective, with an institutional stewardship role in the administration. She reported that there **was no budget allocation for UA activities; however, many related initiatives** framed as composting and environmental education have **received small grants or in-kind contributions** from governmental sources, such as the National Environmental Fund (*Fundo Nacional do Meio Ambiente—FNMA*) and the national savings bank (*Caixa Economica Federal—CEF*), the State Agriculture Secretariat through EPAGRI, and the state water treatment company (*Companhia Catarinense de Águas e Saneamento—CASAN*). Municipal and COMCAP budgets have funded equipment and materials. She noted that community gardens provided opportunities for socialization, particularly to the elderly, and that the **pandemic had a very negative impact** on these activities. However, she emphasized that **this period allowed management to invest in planning before returning to normal activities** in late 2021.

##### Director (Actor 16): Senior management and segmenter

She has been a career civil servant with the city since December 2008, acting as an inspector in the Health Secretariat. She remained in the same role until 2015 when she **took a temporary senior position** in the environmental health surveillance division during a dengue fever outbreak in 2015–2016. She was promoted to director of the Surveillance department of health and environment in early 2020. Since then, she has acted as a supportive liaison between the collaboration participants and senior level management within the Health Secretariat. She also supported the expansion of community gardens with medicinal plants in the Community Health Centers (CHCs) in Florianópolis. She considers that UA has been successfully implemented in Florianópolis since the late 1990s, mostly due to an overall enabling environment, albeit in uncoordinated efforts. Her view is that Health had a critical role in galvanizing many socio-environmental initiatives to support UA. **As a technical staff, she was in touch with vulnerable communities most of her career, contributing with her knowledge to the development of the PMAU policy and participating in the** ***Rede Semear*** **network. In 2020, she was promoted to a managerial position as director and has had marginal contact with the network since then**. Like other interviewees, she noted that the pandemic had a very negative impact on socialization activities in communities, contributing to a fragmentation of the UA movement as a whole.

Regarding the administrative changes beginning in February 2021, she is cautious in advocating for the successful outcomes of institutionalization. **She recognizes that for the PMAU to gain scale, the municipal government needed to take the lead and formalize procedures**. However, **she is unclear about the impacts of this process on the engagement of staff and activists in promoting and implementing the UA policy**.

Table 6 summarizes the collaboration outputs associated with each individual participant. We identified two role identity transition styles associated with their ability to manage multiple roles: integrators and segmenters. We also identified individuals engaged in organization switching (external switching), department switching (internal switching), and no switching during the life cycle of the collaboration.

**Table 6:** Individual participants and their association with collaborative outputs

			Collaborative outputs		
Case	Actor	Role transition style	Activation & Collectivity	Institutionalization	Stability, Decline, Change
Internal switching	1 (Nurse)	Integrator	Urban agriculture and health projects	Medicinal herb gardens	Decline (Lack of power)
	2 (Physician)	Segmenter		Rede Semear	Decline (Lack of personal interest)
	16 (Director)	Segmenter		PMAU policy	Decline (Change of priorities)
External switching	3 (Politician)	Integrator	RdB and CEPAGRO covenant	PMAU and PMAPO policies	Decline (Lack of representation)
	4 (Appointee)	Segmenter		Zero Waste Decree	Change (Zero Waste 2030 Program)
No organization switching	5 (Educator)	Integrator	RdB and PMF covenant	PMAU and PMAPO policies	Decline (Lack of power)
	6 (Manager)	Segmenter		Organizational routines	Decline (Change of priorities)

*RdB* Revolução dos Baldinhos (Bucket Revolution), *CEPAGRO* Centro de Estudos e Promoção da Agricultura de Grupo (NGO working with community agriculture), *PMF* Prefeitura de Florianópolis, *PMAU* Urban Agriculture Policy, *PMAPO* Agroecology and Organics Production Policy

## Discussion

### Individuals Are Central to Understanding Collaborative Governance

**Fig. 4:**
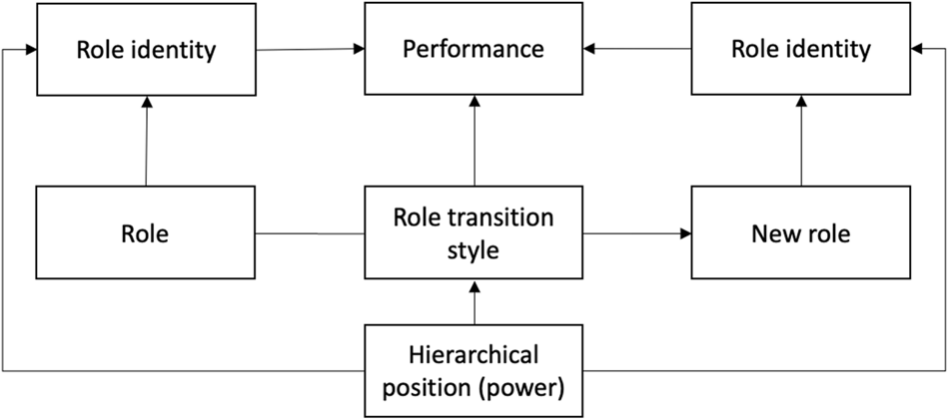
Individuals in collaborative governance over time: A role identity perspective

Figure 4 summarizes the main findings and contributions of this study. Using role identity theory to explore the influence of individual participants on the productivity of collaborative governance, this study shows evidence of how not only role identities influence performance but also how role transition styles and hierarchical power are associated with different outputs along the stages of the development of collaboration.

The literature on understanding individuals in collaborative governance is limited (Bingham and O’Leary 2005; Bryson et al. 2015; Emerson and Nabatchi 2015). Thus, the collaboration process in Florianópolis provides evidence of how individuals change roles and switch hierarchical positions between and within organizations along the trajectory of the collaboration, and how those changes influence the results of the collaboration.

### Individual Role Transitions and the Process Of Collaborative Governance

Initially, we analyzed actors 1 (the nurse), 3 (the politician), and 5 (the educator), associated with the integrator style. Through their multiple role identities, they sought to build collaborations to expand a waste management initiative by integrating composting with other sectors such as UA, health, and education. Table 6 shows how *individuals associated with the integrator style are critical to producing outputs during the activation and collectivity stages of the collaboration*. The flexibility of integrators to overlap activities from their multiple role identities allows them to take and understand different perspectives in collaborative settings. As a result, their participation is essential (1) to discovering different perspectives from diverse actors and (2) to reach a shared motivation to move the collaboration forward (see Emerson and Nabatchi 2015; Tempelaar and Rosenkranz 2019).

Next, we analyzed actors 2 (physician), 4 (political appointee), 6 (manager), and 16 (director). Those individuals are associated with the segmenter style due to different reasons. In fact, according to the expectations of bureaucratic politics and identity theory (Brower and Abolafia 1997; Tempelaar and Rosenkranz 2019), role transitions with high decision-making power from the physician and the political appointee are associated with the segmenter style. More importantly, it is clear how *the segmenter style of these individuals is positively associated with the collaborative outputs during the institutionalization stage of the collaborative life cycle*. They are important to create rules and follow routines that help to scale up existing initiatives. In fact, the establishment of the *Rede Semear* as a type of institutional design, the PMAPO and PMAU laws, the Zero Waste Decree, and organization routines were promoted by the segmenters in our sample.

### Agency of Individuals for Influencing the Productivity of Collaborative Governance

Consitent with previous propositions in the literature (see Ran and Qi 2019), segmenters with decision-making power (e.g., the political appointee and the director) centralize collaboration management to achieve their organizational goals and personal interests, sometimes to the detriment of other partners’ interests. They use their power to deploy the organizational capacity that they control to expand initiatives, giving them political visibility and pleasing politicians to keep segmenters in their highly visible posts or get promotions. The main critique against the Zero Waste 2030 Program was that it moved resources from the UA projects to buy equipment for implementing the Zero Waste policy, a political priority for the politicians in charge. When questioned about the impact of institutionalization on the collaborative effort, an interviewee involved in establishing the *Rede Semear* network commented that UA is a cross-cutting theme that unites diverse interests and implies compromise, listening, and taking these interests into account. “If we overlook other interests, the tendency is to break up the network. People must find an echo of their demands. However, it [*the initiative*] needs to allow accommodation; if it closes too much, it becomes a waste treatment policy. Suppose there is institutionalization without an attempt at colonization. In that case, it can work […] as long as the government does not want to assume hegemony of the process and does not want to colonize other sectors. Colonization would be the government finding itself to be the network’s leader. So, I think it is not institutionalization but the way it is done. The risk is too great if [the government] assumes it owns the initiative. It is not necessarily institutionalization that kills the network; it depends on who has more power.”

Additionally, this study shows that segmenters with decision-making power do not necessarily continue formally participating in the collaboration when their hierarchical role and personal interests change over time. For example, the physician moved from participating in the design of a collaborative network that included the municipality (*Rede Semear*) to leading big data analysis about the COVID-19 pandemic in his new position as Director of the Data Analysis Department.

### Combining Role Identity and Power to Lead Post-institutionalization Stages

Collaborative governance studies discuss whether strong leadership can be a substitute for institutional designs in managing successful collaborations (Douglas et al. 2020; Torfing et al. 2020). This study contributes to theory by providing evidence that inclusive institutional designs and leadership are not substitutes or even sufficient to manage successful collaborations from an individual participants’ perspective due to at least two factors: (1) power differences among participants and (2) conflict between role expectations and transition styles. On the one hand, the lack of decision-making power to implement agreements from individual participants increases coordination costs (Imperial 1999). In this case, the formal participation of integrators declined after the institutionalization of the collaboration due to a lack of decision-making power in their new roles. For example, the nurse declined her formal participation in the initiative once she moved to a department with limited influence on collaborative outcomes. Despite the development of institutional channels to integrate innovation in the initiative (e.g., medicinal herb gardens), the collaboration’s priorities changed from UA projects to waste management with the Zero Waste policy.

On the other hand, the presence of institutional arrangements (e.g., PMAU and PMAPO PMAPO policies) was not enough to manage the UA collaboration in Florianópolis due to the conflict between role expectations and role transition styles. For instance, the councilman struggled with the expectation of his role as a legislator and his tendency to integrate his different role identities (e.g., agronomist, activist, and politician). Partisan politics or superimposing the interests of political parties over the interests of the majority (Mainwaring 1988) do not accommodate the needs of UA groups associated with opposition parties in the policy. As a result, his role in the collaboration is in question, given his unsuccessful activism of UA initiatives for vulnerable populations, as perceived by his voters.

Research using simulations and agent-based modeling (ABM) has previously concluded that the success of the collaboration over time depends on how individual participants manage conflict and adjust their preferences, particularly in contexts with high uncertainty about the best alternative to solve wicked problems (Robertson and Choi 2012; Scott et al. 2019). Additionally, Emerson and Nabatchi (2015) identified different dimensions (e.g., the efficacy of actions/outputs; legitimacy of outcomes) and different units of analysis (e.g., collaboration level, organization level) to evaluate the performance of a collaboration. Results from this study show how individual participants can adjust their preferences by changing priorities based on their agency to influence collaboration. Consequently, collaborations can enter unstable post-institutional stages, in which individual participants decline their participation due to a lack of power to influence the collaboration. Moreover, an unstable post-institutional stage can also be reached when individual participants have the agency to change the focus of the collaboration toward pursuing goals at the organizational level (e.g., amended PMAU; Cultiva Floripa Decree; Zero Waste 2030 Program) instead of joining efforts to catalyze innovative actions (e.g., community waste management, medicinal herb gardens, and urban agriculture and agroecological practices) at the collaboration level.

## Conclusion

The results suggest that individuals do not have fixed organizational roles, and the associated role transition styles have an impact on the productivity of the collaboration. By using identity theory and bureaucratic politics, this study complements the microfoundations of theories from management to understand public organizations. As a result, this study identifies the effects of multiple roles, transition styles, and organizational switching on collaborative governance outputs.

This study adds to the literature on environmental management with implications for theories in public administration. Using pattern matching as a qualitative methodology extends the theory behind collaborative governance by providing evidence on how individual role identities and transition styles affect a collaboration to manage environmental change. The study also demonstrates how these effects are conditional on the development stages throughout the collaboration’s life cycle and the power embedded in the new identity roles after switching organizations/departments. In other words, individual ambidexterity in a collaboration appears to be sequential. In the initial phase, individuals tilt toward exploration but then need to move toward exploitation activities during the institutionalization phase. However, individual participants’ impact on the adaptation requirements typical of complex and uncertain conditions for environmental management needs further study. While this study uses identity theory to analyze the behavior of individuals in collaborative settings, a clear limitation is its focus on organizational roles while not considering other observable and unobservable characteristics that configure the identity of individuals. Future work examining identity in collaborative governance is needed by incorporating gender, ethnicity, religion, and other important sources of identity.

By identifying that not always individual participants have agency for influencing collaborations across different developmental stages, this study emphasizes how power imbalances between individuals can lead toward unstable post-institutional stages: (1) by changing personal priorities due to lack of power to influence the collaboration, and (2) by changing the focus of the collaboration to pursue single organizational goals when individuals have agency to do so.

Further work is needed to test whether individuals with a high level of agency to influence the collaboration, when switching roles, will prioritize the interest of their organizations over collaborative agreements, jeopardizing the stability of the collaboration in the long run due to the impossibility of reconciling policy targets at the organizational and collaborative levels. As environmental management has become highly dependent on collaborative initiatives, complex emerging issues, particularly climate change and biodiversity loss—require understanding that individual roles are important to strengthen collaborative efforts, likewise selecting and preparing individuals to manage complex collaborative development processes as they move from one stage to another.

## Data Availability

Data available upon request.

## Annex 1. Summary of the seven selected actors along the stages of collaborative efforts

*Actor 1. The Nurse, during various stages of development of the collaboration*

**Table:**

Role transition style	Integrator		
Stages	Activation	Institutionalization	Stability, decline, change
Role	Nurse	Department coordinator	Technical area coordinator
Position	Technical staff	Middle management	Technical area coordinator
Impact on the organization	Expanding the scope of the health department to include nutrition, prevention, and social interactions within the Family Health Program (PSF).	Consolidating the city’s role as a policymaker in health and nutrition, adopting UA to enhance the use of herb gardens as prevention practice in the PSF.	Political and administrative changes resulted in weakening UA activities due to the city’s policies to address the COVID-19 pandemic (03/2020).
Impact on Collaboration	Engaging community members and staff/participatory approach to health promotion.	Coordinating participatory actions to expand the initiative in the community and across the city.	She lost power and autonomy in UA activities. The collaboration was also weakened due to the disruption of trust and companionship bonds established along the activation and early institutionalization processes.
Results on project scaling-up	Consolidating experience in health and daycare centers and expanding to other units in the city through participation in internal and external capacity building activities.	Outreach activities helped expand alternative healing practices as prevention throughout the health system in the city. Provided empirical evidence to establish the policy. Participated in the writing up of the decrees.	UA lost priority in the municipal agenda due to the pandemic, which required mobilizing the entire health department, but she is still considered a key actor and spokesperson for the city on the *Cultiva Floripa* program.

*Actor 2. The Physician, during various stages of development of the collaboration*

**Table:**

Role transition style	Segmenter		
Stages	Activation	Institutionalization	Stability, decline, change
Role	Physician—Department A director	Department coordinator on sabbatical	Department B director
Position	Senior management	NA	Senior management
Impact on the organization	Expanding the scope of the health department to include nutrition, prevention, and social interaction. Introduced network governance concept that guided the development and implementation of the multistakeholder UA network.	Leader in *Rede Semear* and health promotion. Paid leave in 2020 due to personal reasons resulted in reduced support to staff and weakened the department.	In a different area, he became less engaged with staff from the previous department, although he continued to participate in the collaboration as an activist. His transition contributed to undermining the department’s health promotion agenda.
Impact on Collaboration	Provided political support to the team by endorsing staff members’ engagement. Contributed to knowledge and leadership in *Rede Semear*.	Loss of leadership and political support inside the administration. It left a vacuum which contributed to weakening the staff’s engagement. This, in turn, had an impact on the collaboration by destabilizing bonds between municipal staff and community members.	He’s no longer involved in the collaboration and is working on big data analysis, mostly directed at pandemic governance.
Results on project scaling-up	Consolidating network governance experience within the municipality (health centers and daycare centers) and expanding to other municipal units through internal and external capacity building activities.	He ceased contributing and supporting the initiative inside the organization and had no impact at this stage.	He contributes marginally by providing data to support the policy and by being part of *Rede Semear*.

*Department A* Sanitary Surveillance and Health Promotion/Municipal Health Secretariat, *Department B* Information Technology and Management/Municipal Health Secretariat

*Actor 3. The Politician during various stages of development of the collaboration*

**Table:**

Role transition style	Integrator		
Stage	Activation	Institutionalization	Stability, decline, change
Role	Agroecology trainer	Councilman	
Position	Senior management		
Impact on the organization	Increasing knowledge about nutrition and health in vulnerable communities. Establishing agroecology as a strategy to address poverty and land tenure issues. Helped galvanize efforts in the city and motivate civil servants to work with community members.	Consolidating urban farming and agroecology as social practices helped the municipality to integrate community action and government routines. As a leader with public support for the UA agenda, he attracted municipal leadership attention to UA. However, being from an opposition party, city staff engaged in this movement were perceived by leadership as adversarial, causing conflicts.	The UA agenda was captured by municipal leadership to get support for the waste management policy. The focus was shifted to improving waste collection while weakening agroecology and community movements.While his role as a politician became more important, his support for his constituency became more limited. Municipal staff was discouraged by leadership to continue participating in activities perceived as politically biased towards his party.
Impact on Collaboration	Engaging community members, leading social movements, providing knowledge, building capacity, and supporting new leadership in communities.	Supporting UA through advocacy and legislation. Strengthening community voice and expanding the UA movement.Has not been able to further support the CSO or broker funding due to political status.	Increasing critical mass in support of municipal legislation and municipal policy. However, having to negotiate with other councilors and the executive branch implied trade-offs that might have negatively impacted his constituency. Being engaged in politics raised issues regarding partisanship and a negative attitude by members of opposition parties in power.
Results on project scaling-up	Consolidating social movements around UA and providing critical mass to support policies.	Promoting UA as a social strategy in Florianópolis’ agenda. Leading Bill of Law and passing legislation on UA, organic farming, and agroecology. Disseminating the healthy nutrition concept in Florianópolis’ school system. Provided empirical evidence for the policy.	Promoting UA and agroecology in Santa Catarina state’s agenda. Provided empirical evidence to establish the policy also at the state level, helping expand UA to other municipalities.

*Actor 4. The Appointee, during various stages of development of the collaboration*

**Table:**

Role transition style	Segmenter		
Stage	Activation	Institutionalization	Stability, decline, change
Role	Superintendent A	Chief Executive Officer COMCAP	Superintendent B
Position	Senior management		
Impact on the organization	Not applicable	Centralized decision-making and operations. Agenda setting: waste mgt and composting. Establishing norms and providing a legal framework at the executive level to connect composting, UA, and zero waste policy.	Administrative reform reduced autarchy’s role, which became only operational. Established superintendence to concentrate budgetary and waste policy decision-making.
Impact on Collaboration	Not applicable	Organizing and strengthening dispersed UA initiatives in the community with policy.	Including composting in the waste management strategy. Engaging the community to produce compost from its own organic waste for the vegetable gardens. Increased bureaucracy, reduced flexibility, and divided producers; the city only pays registered producers for compost.
Results on project scaling-up	Not applicable	Regulation of UA activities, increasing concentration of power and decision-making at the executive level. Increasing bureaucracy and dependency on municipal support but improving organization and selection of candidates for plots and funding.	During the pandemic, developed procedures and routines that led to a division between community-led UA initiatives and municipality policies. Implementing the PMAU tighter norms will make engaging community members harder.

*Superintendent A* Municipal Housing and Sanitation, *Superintendent B* Sanitation, *COMCAP* Waste Management Municipal Autarchy

*Actor 5. The Educator, during various stages of development of the collaboration*

**Table:**

Role transition style	Integrator		
Stage	Activation	Institutionalization	Stability, decline, change
Role	Environmental educator	Environmental educator	Environmental educator
Position	Technical staff	Coordinator	Technical staff
Impact on the organization	Promotes knowledge about the environment, health, and well-being in communities and schools. A strong advocate for UA as a municipal intersectoral program. Encouraging staff participation and actively promoting integration overall.	Strengthened middle mgt engagement, contributed to highlighting the cross-cutting aspect of UA, and helped build capacity on the synergies between UA, environment, health, and nutrition. Helped establish and coordinate the intersectoral group in the municipality promoting public participation. Strengthened the municipality’s role in the community. Lobbying for UA inside the organization. Helped bring to the attention of senior management the political potential of the initiative.	Political and administrative changes resulted in weakening UA activities, mainly due to the city’s policies to address the COVID-19 pandemic, starting on 03/2020. The steering group was dissolved by leadership with the decree.
Impact on Collaboration	As an environmental educator, she promotes knowledge about the environment, health, and well-being in communities and schools. She was also a strong advocate of UA as an intersectoral program in the municipality. Encouraged staff participation and promoted internal and external integration.	She helped improve communication between the parties. The *Cultiva Floripa* program became a reference for the communities that perceive themselves as part of the project. However, they are pressing to be compensated for diverting organic waste from landfills, thus reducing the city’s costs with transport and waste disposal.	Despite their efforts, technical staff hasn’t managed to secure a budget for the initiative. As a result, the collaboration is weakened by the community members’ loss of trust in municipal staff and dissatisfaction with the city. The pandemic made them even more discouraged and demobilized.
Results on project scaling-up	Consolidating experience in health centers and daycare centers.	Outreach activities helped expand the healthy nutrition concept beyond the Florianópolis school system. By increasing public participation, public servants in middle mgt and technical positions gained trust from the community members and staff in other departments.	The change in governance from a participatory to a centralized style reduced the staff’s confidence and agency. Due to the pandemic, UA lost priority in the municipal agenda, and much of the budget, as well as program efforts, were diverted to combat COVID-19 and to waste management measures. Middle management staff is finding other niches to promote UA, as they consider *that Cultiva Floripa* is well established and will overcome these barriers.

*Actor 6. The Manager, during different stages of development of the collaboration*

**Table:**

Role transition style	Segmenter		
Stage	Activation	Institutionalization	Stability, decline, change
Role	Manager/technical advisor	Manager/technical advisor	Senior technical staff
Position	Middle management		Technical staff
Impact on the organization	Supported staff from other departments engaged in UA. Strong UA advocate as an intersectoral program.	Lobbying internally for UA and strengthening the municipality’s role in the community by providing operational support.	She didn’t have a decision-making position and continued to be engaged marginally after her organization was downgraded, from controlling the budget to executing operational activities.
Impact on Collaboration	Facilitated logistics and intermediated communication with senior management.	Supported staff from other departments engaged in UA. Facilitated logistics and intermediated communication with senior management.	Informal facilitator role in the collaboration was reduced, but she continued participating marginally.
Results on project scaling-up	Strengthened the network by enabling operational support.	Strengthened the network by enabling operational support.	Informal facilitator role in the collaboration was reduced, but she continued participating marginally.

*Actor 16. The Director, during different stages of development of the collaboration*

**Table:**

Role transition style	Segmenter		
Stage	Activation	Institutionalization	Stability, decline, change
Role	Manager	Manager	Director
Position	Middle management	Middle management	Senior management
Impact on the organization	Participated in her department’s activities involving development of the UA policy.	Lobbying internally for UA and strengthening the municipality’s role in the community by providing operational support.	She didn’t have a decision-making position and continued to be engaged marginally after her organization was downgraded, from controlling the budget to executing operational activities.
Impact on Collaboration	Participated in drafting the PMAU and contributed knowledge about health risks associated with waste dumping. Strengthening critical mass.	Supported staff from the Health Secretariat to engage in UA.	As an administrative staff, she facilitates the dialog between her team and senior management in support of the UA policy.
Results on project scaling-up	Strengthened the network by contributing her knowledge to the PMAU.	Promoted the integration of the different health departments in the PMAU activities regarding medicinal plant gardens in the community health centers (CHCs).	Continued to promote the role of the Health Secretariat in implementing the PMAU. As a senior manager, she supported the expansion of the community gardens in the CHCs.

## Footnotes

1 Expected patterns from theory are similar to traditional hypothesis testing. The main difference is that “pattern matching encourages the use of more complex or detailed hypotheses and treats the observations from a multivariate rather than a univariate perspective” (Trochim, 1989, p. 357).
